# Long-Term Pancreatic Beta Cell Exposure to High Levels of Glucose but Not Palmitate Induces DNA Methylation within the Insulin Gene Promoter and Represses Transcriptional Activity

**DOI:** 10.1371/journal.pone.0115350

**Published:** 2015-02-06

**Authors:** Kota Ishikawa, Shin Tsunekawa, Makoto Ikeniwa, Takako Izumoto, Atsushi Iida, Hidetada Ogata, Eita Uenishi, Yusuke Seino, Nobuaki Ozaki, Yoshihisa Sugimura, Yoji Hamada, Akio Kuroda, Keiko Shinjo, Yutaka Kondo, Yutaka Oiso

**Affiliations:** 1 Department of Endocrinology and Diabetes, Nagoya University Graduate School of Medicine, 65 Tsurumai-cho, Showa-ku, Nagoya, 466–8550, Japan; 2 Department of Oral and Maxillofacial Surgery, Nagoya University Graduate School of Medicine, Nagoya, Japan; 3 Department of Metabolic Medicine, Nagoya University Graduate School of Medicine, Nagoya, Japan; 4 Diabetes Therapeutics and Research Center, The University of Tokushima, Tokushima, Japan; 5 Department of Epigenomics, Nagoya City University Graduate School of Medical Sciences, Nagoya, Japan; University of Nantes, FRANCE

## Abstract

Recent studies have implicated epigenetics in the pathophysiology of diabetes. Furthermore, DNA methylation, which irreversibly deactivates gene transcription, of the insulin promoter, particularly the cAMP response element, is increased in diabetes patients. However, the underlying mechanism remains unclear. We aimed to investigate insulin promoter DNA methylation in an over-nutrition state. INS-1 cells, the rat pancreatic beta cell line, were cultured under normal-culture-glucose (11.2 mmol/l) or experimental-high-glucose (22.4 mmol/l) conditions for 14 days, with or without 0.4 mmol/l palmitate. DNA methylation of the rat insulin 1 gene (*Ins1*) promoter was investigated using bisulfite sequencing and pyrosequencing analysis. Experimental-high-glucose conditions significantly suppressed insulin mRNA and increased DNA methylation at all five CpG sites within the *Ins1* promoter, including the cAMP response element, in a time-dependent and glucose concentration-dependent manner. DNA methylation under experimental-high-glucose conditions was unique to the *Ins1* promoter; however, palmitate did not affect DNA methylation. Artificial methylation of *Ins1* promoter significantly suppressed promoter-driven luciferase activity, and a DNA methylation inhibitor significantly improved insulin mRNA suppression by experimental-high-glucose conditions. Experimental-high-glucose conditions significantly increased DNA methyltransferase activity and decreased ten-eleven-translocation methylcytosine dioxygenase activity. Oxidative stress and endoplasmic reticulum stress did not affect DNA methylation of the *Ins1* promoter. High glucose but not palmitate increased ectopic triacylglycerol accumulation parallel to DNA methylation. Metformin upregulated insulin gene expression and suppressed DNA methylation and ectopic triacylglycerol accumulation. Finally, DNA methylation of the *Ins1* promoter increased in isolated islets from Zucker diabetic fatty rats. This study helps to clarify the effect of an over-nutrition state on DNA methylation of the *Ins1* promoter in pancreatic beta cells. It provides new insights into the irreversible pathophysiology of diabetes.

## Introduction

Type 2 diabetes is an insulin insufficiency state caused by decreased pancreatic beta cell function and mass [1,2]. Genetic and environmental factors influence the development of type 2 diabetes, with the nutritional state being particularly important. In preclinical type 2 diabetes, beta cells secrete excessive insulin and considerably expand their mass to compensate for the increased metabolic load and obesity-associated insulin resistance. However, failure of beta cell adaptation leads to type 2 diabetes onset with declining insulin secretion and beta cell mass [1]. Beta cell dysfunction then deteriorates, particularly in individuals with poor glycemic control, and eventually becomes irreversible despite glucotoxicity treatments providing temporary improvements in the dysfunction to some extent [2].

When glycemic control is poor, it is widely accepted that the associated diabetic complications will worsen. Moreover, the Diabetes Control and Complications Trial/Epidemiology of Diabetes Interventions and Complications and United Kingdom Prospective Diabetes Study showed correlations between transient poor glycemic control and progression of diabetic complications [3,4]. This “metabolic memory” or “legacy effect” phenomenon is partially regulated by epigenetic modification, which causes histone 3 lysine 4 monomethylation in aortic endothelial cells under transient high-glucose states and sustains the high inflammatory cytokine levels under subsequent normoglycemia [5–7].

Epigenetic modification regulates gene expression without altering the DNA sequence and mainly occurs through histone modification and DNA methylation [8]. Histone modifications usually control the chromatin structure and transcriptional activity and include methylation, acetylation, phosphorylation, sumoylation, and ubiquitination at histone N-terminals [9]. DNA methylation occurs at the cytosine site in the CpG dinucleotide where it irreversibly deactivates gene transcription and is balanced by the effects of DNA methyltransferase (DNMT) and ten-eleven-translocation methylcytosine dioxygenase (TET) [10]. DNA methylation represses transcriptional activity, either by directly preventing transcriptional factors from binding to their cognate sequences or by recruiting transcriptional repressor complexes that form heterochromatin (“closed,” or inactive, chromatin) [11].

Recent studies have reported that epigenetic modulation of beta cells could be of pathogenic importance in type 2 diabetes. A genome-wide DNA methylation analysis identified different DNA methylation patterns on candidate genes in the islets of patients with type 2 diabetes. In that study, 17 of 40 type 2 diabetes candidate genes were differently methylated, e.g. potassium voltage-gated channel KQT-like subfamily member 1 (*KCNQ1*) and transcription factor 7-like 2 (*TCF7L2*). Moreover, functional analysis demonstrated that cyclin-dependent kinase inhibitor 1A (*CDKN1A*) and phosphodiesterase 7B (*PDE7B*), which exhibit decreased DNA methylation and increased gene expression in type 2 diabetes, result in impaired insulin secretion and exocyst complex component 3-like 2 (*EXOC3L2*), which exhibits increased DNA methylation and decreased gene expression in type 2 diabetes, results in decreased exocytosis from pancreatic beta cells [12]. DNA methylation of the pancreatic and duodenal homeobox factor-1 (*Pdx1*) promoter of intrauterine growth retardation rats is considered a major cause of susceptibility to glucose intolerance in adulthood [13]. A recent study involving patients with type 2 diabetes showed that elevated DNA methylation of the insulin gene promoter, particularly at the cAMP response element (CRE) site, was proportional to HbA_1c_ levels and inversely proportional to insulin gene expression [12,14]. However, the precise mechanism underlying DNA methylation in the diabetic state remains unclear.

We hypothesized that long-term environmental exposure to high glucose levels would cause epigenetic modification and irreversible damage to beta cells. This study aimed to elucidate the effects of an over-nutrition state on epigenetic modification in the insulin gene promoter. In particular, we investigated the role of the high glucose state on DNA methylation of the CpG site in the insulin gene promoter.

## Materials and Methods

### Materials

Sodium palmitate, forskolin, 3-isobutyl-1-methylxanthine (IBMX), 5-Aza-2′-deoxycytidine (DAC), and N-acetyl-cysteine were obtained from Sigma (St Louis, MO, USA). Metformin was obtained from Enzo Life Sciences (Farmingdale, NY, USA). Hydrogen peroxide (H_2_O_2_) was obtained from Santoku Chemical Industries (Tokyo, Japan). Thapsigargin and tauroursodeoxycholic acid (TUDCA) were obtained from Calbiochem (La Jolla, CA, USA). The insulin enzyme-linked immunosorbent assay (ELISA) kit was obtained from Morinaga (Tokyo, Japan), and the triglyceride quantification colorimetric/fluorometric kit was obtained from BioVision (Milpitas, CA, USA). EpiQuik DNMT activity/inhibition assay and Epigenase 5 mC-Hydroxylase TET activity/inhibition assay kits were obtained from Epigentek (Farmingdale, NY, USA). The Cell Proliferation Kit I (MTT assay) was obtained from Roche Applied Science (Branford, CT, USA).

### Cell culture

The pancreatic beta cell line (INS-1 cells) were provided by Dr. CB Wollheim (University of Geneva, Geneva, Switzerland) [15]. They were cultured in RPMI1640 media supplemented with 10% fetal bovine serum (FBS), 2 μl/500 ml beta-mercaptoethanol, and antibiotics (100 units/ml penicillin–100 μg/ml streptomycin). Cells were maintained at 37°C in a humidified atmosphere containing 95% air and 5% CO_2_. Cells were passaged by trypsinization and were subcultured every fourth day. Cells (passage: 45–70) were cultured under the conditions indicated for each experiment.

Palmitate was precomplexed to FFA-free bovine serum albumin (BSA) (Wako Pure Chemical Industries, Japan) at a 2:1 (palmitate:BSA) molar ratio. Control cells were incubated with media containing FFA-free BSA at the same concentration as palmitate-exposed cells.

### Animals

Male Zucker diabetic fatty rats (ZDF rats; Charles River Laboratories, Wilmington, MA, USA), a diabetes-prone model due to a mutated leptin receptor, were maintained in a 12-h light/dark cycle with free access to water and food (Purina Diet 5008, Charles River Laboratories). All research procedures involving animals were performed in accordance with the Laboratory Animals Welfare Act, the Guide for the Care and Use of Laboratory Animals, and the Guidelines and Policies for Rodent Experiments provided by the Institutional Animal Care and Use Committee at the Nagoya University Graduate School of Medicine and were reviewed and approved by the Institutional Animal Care and Use Committee. The protocol was approved by the committee on the Ethics of Animal experiments of the Nagoya University Graduate School of Medicine (Permit Number: 26060). All surgeries were performed under sodium pentobarbital anesthesia, and reasonable efforts were made to minimize suffering. Rats were sacrificed by intraperitoneal administration of sodium pentobarbital (200 mg/kg).

### Real-time polymerase chain reaction (PCR)

Total RNA was extracted from INS-1 cells using the RNeasy Plus Mini kit from Qiagen (Valencia, CA, USA). Target gene mRNA expression relative to phosphatidylinositol 3-kinase (*Pi3k*) p85 was quantified using the Power SYBR Green RNA-to-CT 1-Step kit in the 7300 Real-Time PCR System (Applied Biosystems, Foster City, CA, USA). The sequences of the specific primer pairs are described in S1 Table.

### Pyrosequencing analysis

Nucleotide sequences for the rat *Ins1* gene (Gene ID: 24505) and insulin receptor substrate 2 (*Irs2*) gene (Gene ID: 29376) were obtained from GenBank. Genomic DNA was extracted from INS-1 cells and rat pancreatic islets using the DNeasy tissue kit (Qiagen). Extracted DNA (2 μg) was then subjected to bisulfite conversion using the EpiTect Bisulfite kit (Qiagen). Bisulfite-treated DNA (1 μl) was amplified by the universal primer approach in 50 μl reaction mixture containing primers and 0.2 U rTaq polymerase from Takara (Otsu, Japan) [16]. Primers for pyrosequencing analysis were designed using Pyrosequencing Assay Design software (Biotage, Westborough, MA, USA). The biotinylated PCR products by universal primer approach were immobilized with streptavidin-coated Sepharose beads, purified, and then denatured using a 0.2 mol/l NaOH solution. The purified single-stranded PCR products were annealed to 0.3 μmol/l pyrosequencing primers, and pyrosequencing was performed on Biotage’s PSQ 96 MA Pyrosequencing System. Following this, the methylation rate was calculated using Qiagen’s PyroMark CpG software. The primer sequences for pyrosequencing analysis and PCR are described in S2 Table.

### Bisulfite sequencing analysis

The rat *Ins1* gene was amplified with pairs of gene-specific primers (S3 Table) in a mixture containing bisulfite-treated DNA (100 ng). PCR was performed using TaKaRa EpiTaq HS (Takara). The bisulfite-PCR product of the rat *Ins1* promoter was cloned into the pGEM-T Easy Vector (Promega, Madison, WI, USA) and sequenced with a T7 primer (Takara Dragon Genomics Center, Mie, Japan). At least 30 clones were sequenced per sample.

### Luciferase assay

INS-1 cells were transfected with a pGL4.10 [*luc2*] vector containing rat *Ins1* 469-base pair (bp) promoter and pGL4.74 [*hRluc*/TK] vector using the FuGENE HD reagent (Promega) according to the manufacturer’s protocol. The pGL4.10 [*luc2*] vector (Promega, Madison, WI) was digested with *Bgl*II/*Hind*III and treated with alkaline phosphatase (CIP) (New England BioLabs, Ipswich, MA). A fragment of the rat *Ins1* promoter (−304 to +192 bp containing five CpG sites) was amplified by PCR using genomic DNA and primers that added *Bgl*II and *Hind*III sites to the ends. PCR products were inserted into the pGEM-T Easy Vector (Promega) and amplified in SOC medium (super optimal broth with catabolite repression). The plasmid sequences were confirmed by DNA sequencing. The cloned promoter fragments were excised and subcloned upstream of the firefly luciferase gene in the pGL4.10 [*luc2*] vector using T4 DNA Ligase (Promega) according to the manufacturer’s recommendation and transformed into DH5α competent cells (Promega) for plasmid production. The pGL4.10 [*luc2*] vector containing the rat *Ins1* 469-bp promoter was either methylated using 10 U of M.*Sss*I CpG methyltransferase (New England BioLabs) or mock-methylated in a parallel control reaction without the enzyme. Luciferase activity was measured 48 h after transfection. Firefly and *Renilla* luciferase activities in cell lysates were measured using the Dual-Luciferase Reporter Assay System (Promega) in a Lumat LB 9507 luminometer (Berthold Technologies, Bad Wildbad, Germany) according to the manufacturer’s instruction. Firefly luminescence was normalized by the *Renilla* luminescence.

### Glucose-stimulated insulin secretion (GSIS)

INS-1 cells were preincubated with 2.8 mmol/l Krebs–Ringer buffer (KRB) buffer for 30 min and stimulated with 16.7 mmol/l glucose for 30 min. We measured supernatant as release and acid–ethanol extract as content. Release and content were measured using H.T.R.F (Cisbio Bioassays, France). The amount of insulin secreted was normalized by cellular insulin contents.

### Pancreatic islet isolation

Pancreatic islets were isolated from 12-week-old ZDF rats by collagenase digestion, as described previously [17].

### Measurement of the insulin content of isolated islet

The total pancreatic insulin content was measured according to a standard acid–ethanol extraction protocol. The islet insulin was measured using H.T.R.F. The amount of insulin secreted was normalized by pancreatic weight.

### Immunofluorescence staining

For morphometric analysis, pancreatic islets were isolated from 12-week-old male ZDF rats. The pancreas was fixed in 4% paraformaldehyde and sequentially washed thoroughly in phosphate-buffered saline containing 10% and 20% sucrose. They were then embedded in OCT Compound (Sakura Finetek, Tokyo, Japan) and frozen. Serial 10-μm sections were cut at 100-μm intervals, and five sections were randomly selected from each pancreas. The sections were incubated overnight with polyclonal anti-insulin guinea pig antibody and polyclonal anti-glucagon rabbit antibody (1:500) from Abcam (Tokyo, Japan) at 4°C. After washing with phosphate-buffered saline, they were incubated for 1 h in a mixture of rhodamine-conjugated anti-guinea pig and anti-rabbit immunoglobulin G antibody before being incubated with 4′,6-diamidino-2-phenylindole (DAPI) solution (1:2000; Dojindo (Tokyo, Japan) for 20 min. The sections were analyzed using the BZ-9000 Fluorescent Microscope System from Keyence (Osaka, Japan). The ratio of glucagon-positive cells to insulin-positive cells was calculated using the HS BZ-II analysis application (Keyence). In total, 90 islets from three rats were estimated per group.

### Statistical analysis

Data are expressed as mean ± standard error along with experiment numbers. Differences between the means of the two groups were compared by unpaired two-tailed Student *t* test (Microsoft Excel 2010). Comparison of quantitative variables among groups were performed using analysis of variance (ANOVA) with the Tukey post-hoc test via GraphPad Prism (v.6.03; GraphPad Software, San Diego, CA, USA). We considered *p* values ≤ 0.05 to be statistically significant.

## Results

### Glucotoxicity and DNA methylation of the CpG site at CRE in the *Ins1* promoter

**Fig 1:**
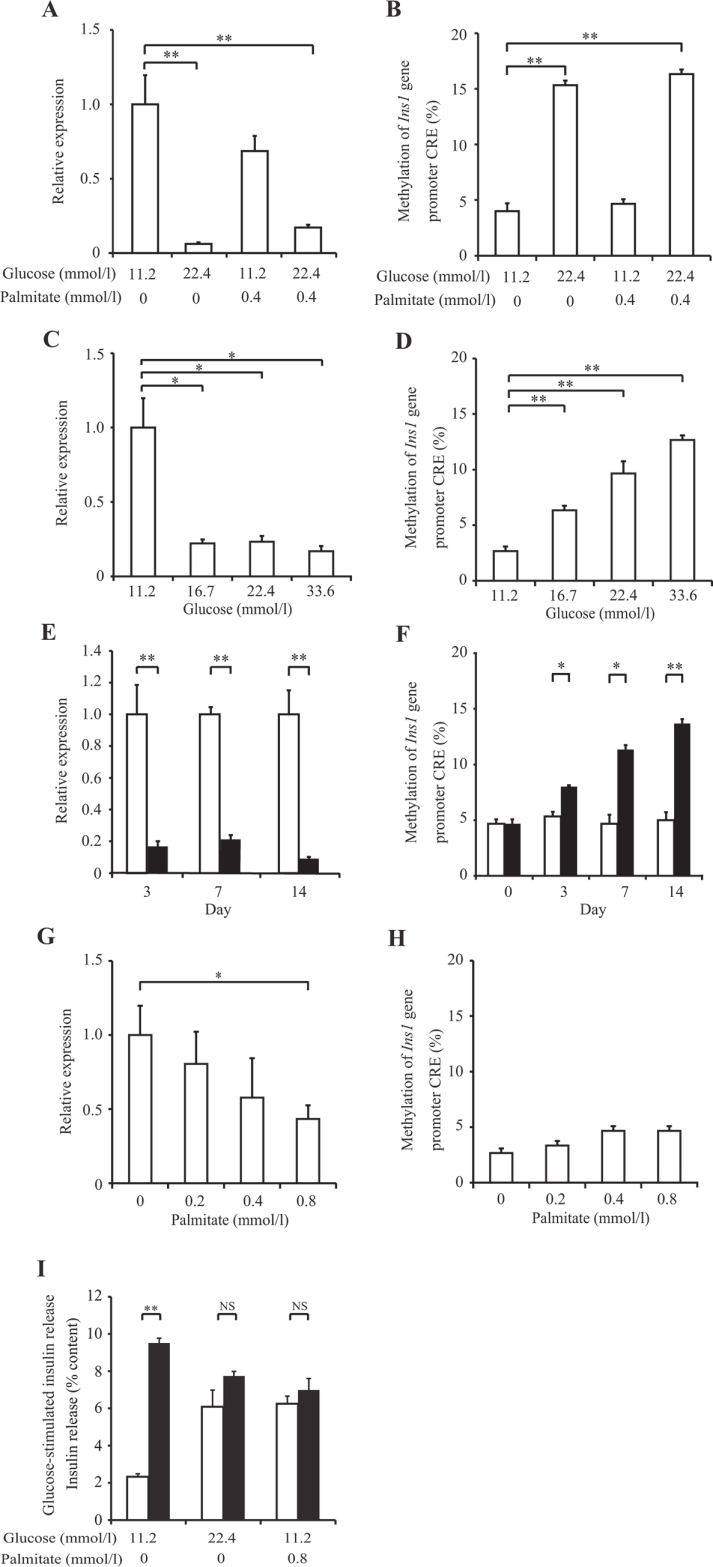
Insulin mRNA levels and DNA methylation of the *Ins1* promoter in high-glucose conditions. (A–D) INS-1 cells were cultured for 14 days. (E and F) under normal-culture-glucose (11.2 mmol/l; white bar) or experimental-high-glucose (22.4 mmol/l; black bar) conditions. (G and H) INS-1 cells cultured in 11.2 mmol/l glucose conditions with palmitate for 14 days. Insulin mRNA levels (A, C, E, and G) were examined by real-time PCR analysis. DNA methylation of the *Ins1* promoter (B, D, F, and H) was examined by pyrosequencing analysis. (I) INS-1 cells were cultured for 14 days under the indicated conditions. Following this, GSIS was performed with low glucose (2.8 mmol/l; white bar) or high glucose (16.7 mmol/l; black bar) for 30 min. All results are mean ± SEM (*n* ≥ 4). Asterisks indicate statistically significant differences (*p < 0.05, **p < 0.01).

Insulin transcription is mainly regulated by the promoter region located approximately 400 nucleotides upstream of the transcription start site [18]. Both rat insulin 1 (*Ins1*) and insulin 2 (*Ins2*) genes have one CRE site in their promoters. The CRE site in the *Ins1* promoter but not in the *Ins2* promoter has a CpG dinucleotide sequence susceptible to DNA methylation [19] (S1 Fig.). Therefore, we examined the DNA methylation of the CpG site at CRE in the *Ins1* promoter (which we refer to as DNA methylation of the *Ins1* promoter) in an over-nutrition state. INS-1 cells were cultured under either normal-culture-glucose (NG; 11.2 mmol/l) or 22.4 mmol/l experimental-high-glucose (HG) conditions for 14 days with or without 0.4 mmol/l palmitate. Incubation under HG conditions, with and without 0.4 mmol/l palmitate, significantly decreased insulin mRNA levels by 80%–95% compared with that under NG conditions (*p* < 0.01). NG plus palmitate did not change insulin mRNA levels (Fig. 1A). Similarly, HG with and without palmitate also markedly increased DNA methylation of the *Ins1* promoter, although palmitate did not influence DNA methylation under NG conditions (NG, 4% ± 0.4%; NG plus palmitate, 4.6% ± 0.4%; HG, 15.3% ± 0.8%; HG plus palmitate, 16.3% ± 0.4%; *p* < 0.01; Fig. 1B). Next, we evaluated DNA methylation of the *Irs2* gene, which has a CRE site in a CpG island of its promoter [20] (S1 Fig.), to confirm whether the effect of HG conditions on DNA methylation was specific to the *Ins1* promoter. None of the conditions affected either DNA methylation at the CRE site of the *Irs2* promoter or *Irs2* mRNA levels (S2 Fig.).

Following this, we investigated the effect of glucose concentrations and incubation periods on DNA methylation of the *Ins1* promoter in INS-1 cells. Insulin mRNA levels significantly decreased by 75%–85% under 16.7, 22.4, and 33.6 mmol/l glucose conditions (Fig. 1C), and DNA methylation of the *Ins1* promoter significantly increased compared with that under NG conditions (6.3% ± 0.4%, 9.7% ± 1.1%, 12.7% ± 0.4%, and 2.7% ± 0.4%, respectively; Fig. 1D). Insulin mRNA levels significantly decreased by 80%–90% at days 3, 7, and 14 (*p* < 0.01) (Fig. 1E), and DNA methylation of the *Ins1* promoter significantly increased at days 7 and 14 under HG conditions compared with that under NG conditions (day 3, 5.0% ± 0.7%; day 7, 7.3% ± 0.4%; day 14, 12.3% ± 0.4%; Fig. 1F). NG plus 0.8 mmol/l palmitate caused insulin mRNA levels to decease by 55%, whereas DNA methylation of the *Ins1* promoter did not increase (Fig. 1G and 1H). After 14 days of culture under 22.4 mmol/l HG or NG plus 0.8 mmol/l palmitate, GSIS of these cells was significantly decreased.

These data show that long-term incubation in the HG state (glucotoxicity) rather than palmitate toxicity is essential for DNA methylation of the *Ins1* promoter. In beta cells, DNA methylation caused by HG was not global because no DNA methylation occurred at the CRE site of the *Irs2* promoter under the HG state. DNA methylation by glucotoxicity was both time and concentration dependent.

### Gene transcription suppressed by DNA methylation of the *Ins1* promoter

**Fig 2:**
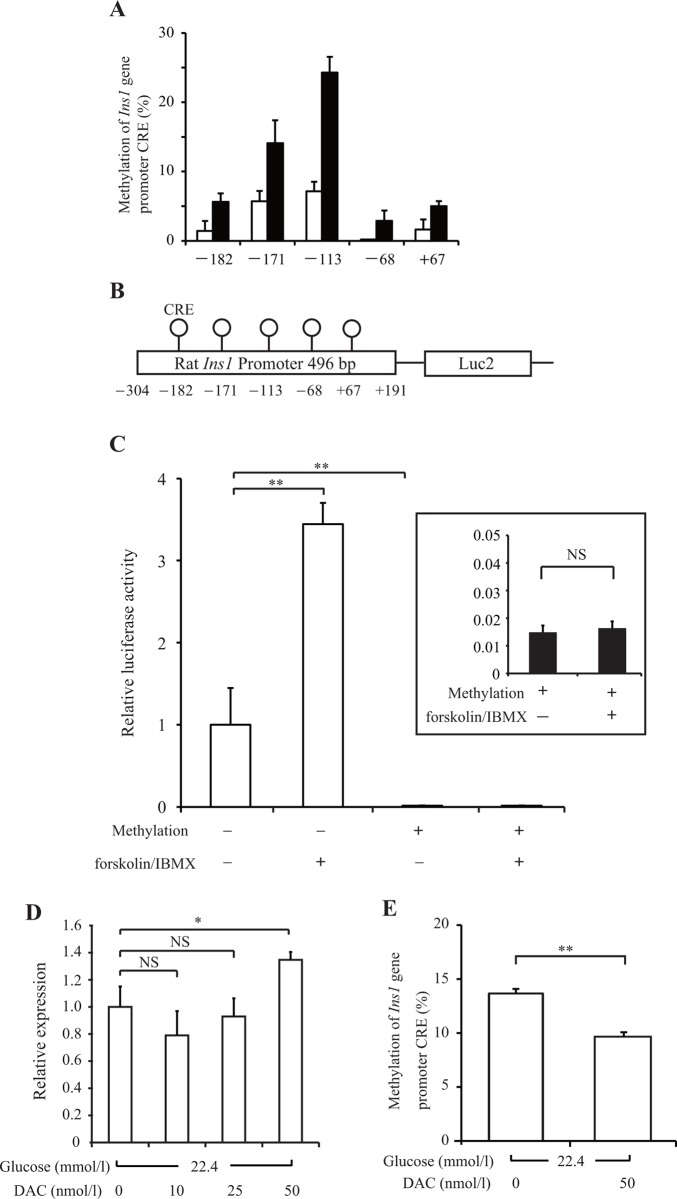
The contribution of DNA methylation of the *Ins1* promoter. (A) INS-1 cells were cultured under normal-culture-glucose (11.2 mmol/l; white bar) or experimental-high-glucose (22.4 mmol/l; black bar) conditions for 14 days. DNA methylation of the *Ins1* promoter was examined by bisulfite sequencing analysis. (B) A diagram of the 496-bp rat *Ins1* promoter (position −304 to +191 bp relative to the transcription start site) in luciferase reporter plasmids. The positions of CpG sites are represented by lollipop markers. (C) Methylated (black bar) or mock-methylated (white bar) rat *Ins1* promoter-transfected INS-1 cells were incubated at 5.6 mmol/l glucose with/without cAMP-increasing agents, 1 μmol/l forskolin and 10 μmol/l IBMX (forskolin/IBMX), for 3 h. Luciferase activities are presented as relative expression compared with the mock-methylated vectors without forskolin/IBMX stimulation. The inset shows a magnified image of the methylated vector. (D and E) INS-1 cells were treated with 5-Aza-2′-deoxycytidine (DAC) for the last 3 days of the 14-day incubation under 22.4 mmol/l high glucose conditions, and the medium containing DAC was changed every 24 h. Insulin mRNA levels (D) were examined by real-time PCR. DNA methylation of the *Ins1* promoter (E) was examined by pyrosequencing analysis. All results are mean ± SEM (*n* ≥ 4). Asterisks indicate statistically significant differences (*p < 0.05, **p < 0.01).

In addition to the CpG site of CRE, the rat *Ins1* promoter contains four other CpG sites (−171, −113, −68, and +67) within a 500-bp region upstream of the ATG start codon. To confirm whether long-term HG incubation specifically induced DNA methylation at the CRE site of interest, we evaluated DNA methylation at the other sites. Bisulfite sequencing analysis revealed that long-term HG incubation induced DNA methylation at all CpG sites within the rat *Ins1* promoter (Fig. 2A).

Because our insulin primer cannot distinguish between *Ins1* and *Ins2*, we then examined *Ins1* promoter activity using a luciferase assay in the pGL4.10 vector with methylated or mock-methylated *Ins1* 469-bp promoter sequences to estimate the direct relationship between the DNA methylation of the *Ins1* promoter and gene transcription (Fig. 2B). As shown in Fig. 2C, compared with the mock-methylated vector, the methylated rat *Ins1* 469-bp promoter suppressed luciferase activity by 95% (*p* < 0.01). In the mock-methylated vector, luciferase activity was increased approximately threefold by cAMP stimulation for 3 h with 1 μmol/l forskolin/10 μmol/l IBMX (*p* < 0.01). Meanwhile, the response to cAMP stimulation in the methylated vector completely disappeared (Fig. 2C).

Following this, the DNA methylation inhibitor DAC was used to estimate the deleterious effect of glucotoxicity on insulin gene expression via DNA methylation. INS-1 cells under 22.4 mmol/l HG conditions were treated with the indicated concentrations of DAC for the last 3 days of the 14-day incubation period (Fig. 2D). We found that 50 nmol/l DAC significantly decreased DNA methylation of the *Ins1* promoter (*p* < 0.01) and improved insulin mRNA suppression under HG conditions (*p* < 0.05) (Fig. 2D and 2E). These data suggest a direct relationship between DNA methylation of the *Ins1* promoter and insulin gene transcription that is induced by glucotoxicity.

### Glucotoxicity increased DNMT activity and decreased TET activity

**Fig 3:**
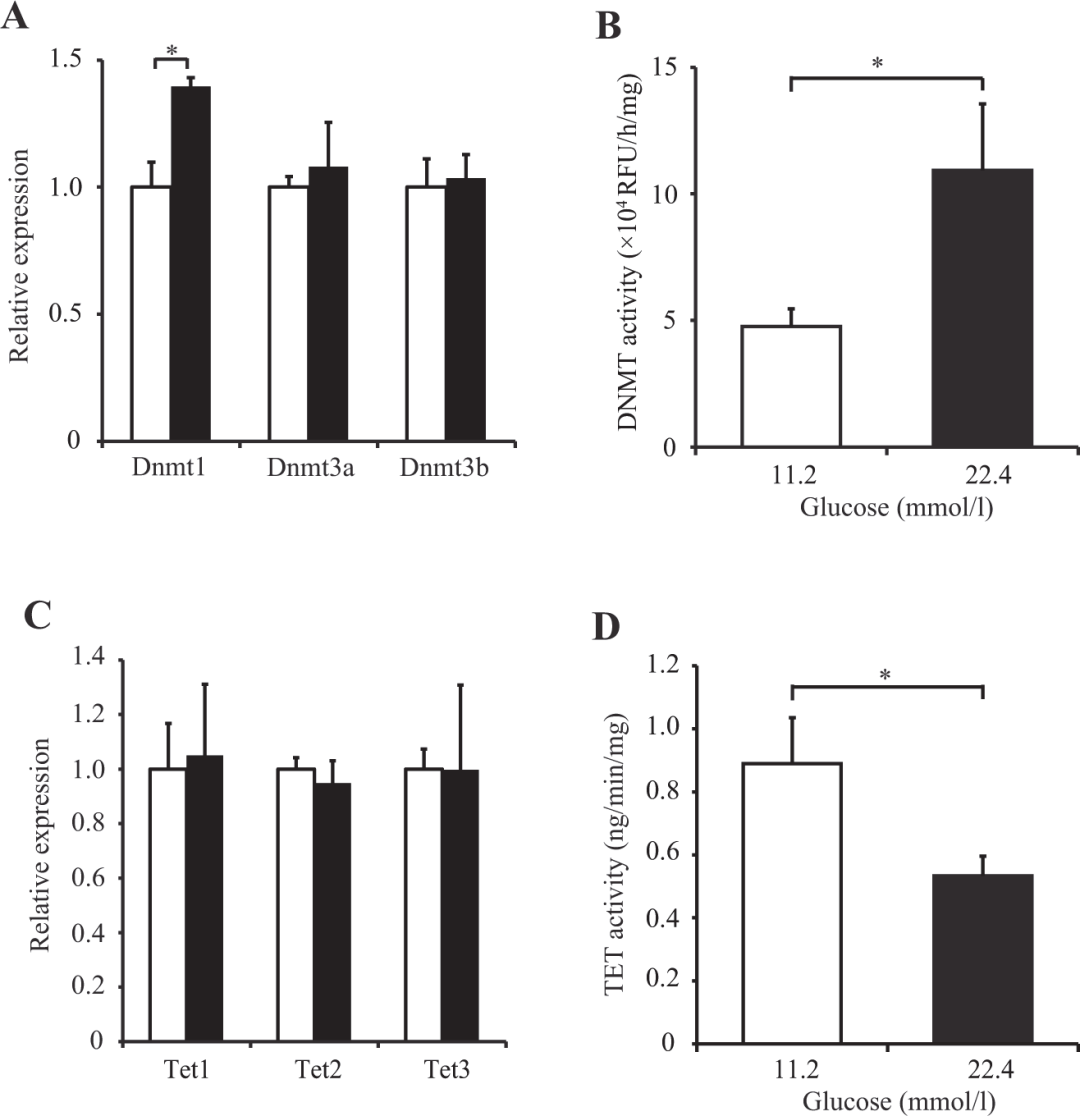
The effect of a high-glucose state on DNMT and TET in INS-1 cells. (A-D) INS-1 cells were cultured under normal-culture-glucose (11.2 mmol/l; white bar) or experimental-high-glucose (22.4 mmol/l; black bar) conditions for 14 days. The *Dnmt* (A) and *Tet* (C) mRNA levels were examined by real-time PCR. DNA methyltransferase (DNMT) (B) and ten-eleven-translocation methylcytosine dioxygenase (TET) (D) activities were examined by ELISA. All results are mean ± SEM (*n* ≥ 4). Asterisks indicate statistically significant difference (*p < 0.05, **p < 0.01).

We evaluated the effect of glucotoxicity on DNA methylation through the DNA methylation modulators DNMT and TET in INS-1 cells. DNMT exists in three isoforms; DNMT1 maintains the methylation pattern during cell replication and DNMT 3a and 3b lead to de novo DNA methylation. TET also exists in three isoforms, TET1, TET2, and TET3, and catalyzes demethylation depending on α-ketoglutarate (αKG) and iron (II) oxide. Compared with NG conditions, significant increases in both *Dnmt1* mRNA levels (*p* < 0.05) and DNMT activity (twofold increase; *p* < 0.05) were observed under 22.4 mmol/l HG conditions for 14 days (Fig. 3A and 3B). Compared with NG conditions, *Tet1*, *Tet2*, and *Tet3* mRNA levels did not change, but TET activity decreased by 50% under the same HG conditions (*p* < 0.05) (Fig. 3C and 3D). These data suggest that glucotoxicity upregulates methylation mechanisms through increased DNMT activity and downregulates demethylation mechanisms through decreased TET activity.

### Oxidative stress and endoplasmic reticulum (ER) stress

**Fig 4:**
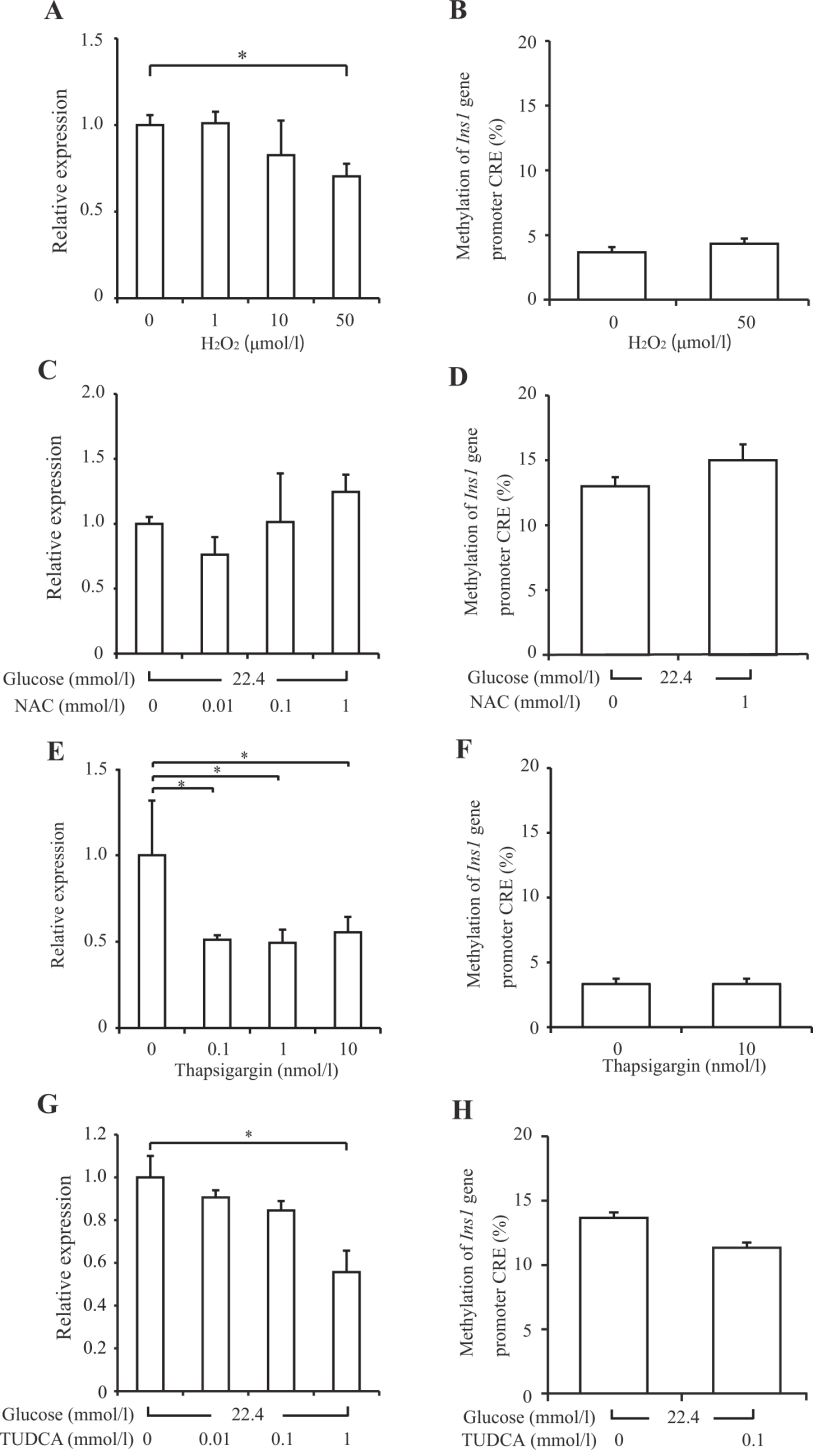
Oxidative stress and endoplasmic reticulum (ER) stress did not induce DNA methylation of *Ins1* promoter. INS-1 cells were cultured for 14 days under the following conditions: (A and B) with H_2_O_2_ in 11.2 mmol/l glucose; (C and D) with N-acetyl-cysteine (NAC) in 22.4 mmol/l glucose; (E and F) with thapsigargin in 11.2 mmol/l glucose; and (G and H) with tauroursodeoxycholic acid (TUDCA) in 22.4 mmol/l glucose. Insulin mRNA levels (A, C, E, and G) were examined by real-time PCR. DNA methylation of the *Ins1* promoter (B, D, F, and H) was examined by pyrosequencing analysis. All results are means ± SEM (*n* ≥ 4). Asterisks indicate statistically significant differences (*p < 0.05, **p < 0.01).

Next, we evaluated the effects of oxidative stress and ER stress, the putative mechanisms through which glucotoxicity affects DNA methylation of the *Ins1* promoter. Treatment of INS-1 cells with 50 μmol/l H_2_O_2_ (an oxidative stress inducer) for 14 days significantly decreased insulin mRNA levels by 30% without changing DNA methylation of the *Ins1* promoter (*p* < 0.05) (Fig. 4A and 4B). Treatment of INS-1 cells with 1 mmol/l N-acetyl-cysteine (an antioxidant agent) for 14 days influenced neither the decreased insulin mRNA nor the elevated DNA methylation induced by 22.4 mmol/l HG conditions (Fig. 4C and 4D).

Treatment of INS-1 cells with 10 nmol/l thapsigargin (an ER stress inducer) for 14 days significantly decreased insulin mRNA levels by 45% without changing DNA methylation of the *Ins1* promoter (*p* < 0.05) (Fig. 4E and 4F). INS-1 cell treatment with 0.1 mmol/l TUDCA (a chemical chaperone that improves protein-folding capacity) for 14 days influenced neither the decreased insulin mRNA nor the elevated DNA methylation of the *Ins1* promoter induced under 22.4 mmol/l HG conditions (Fig. 4G and 4H). Thus, neither oxidative stress nor ER stress induced DNA methylation of the *Ins1* promoter.

### DNA methylation of the *Ins1* promoter and intracellular triacylglycerol (TAG) under HG conditions and metformin treatment

**Fig 5:**
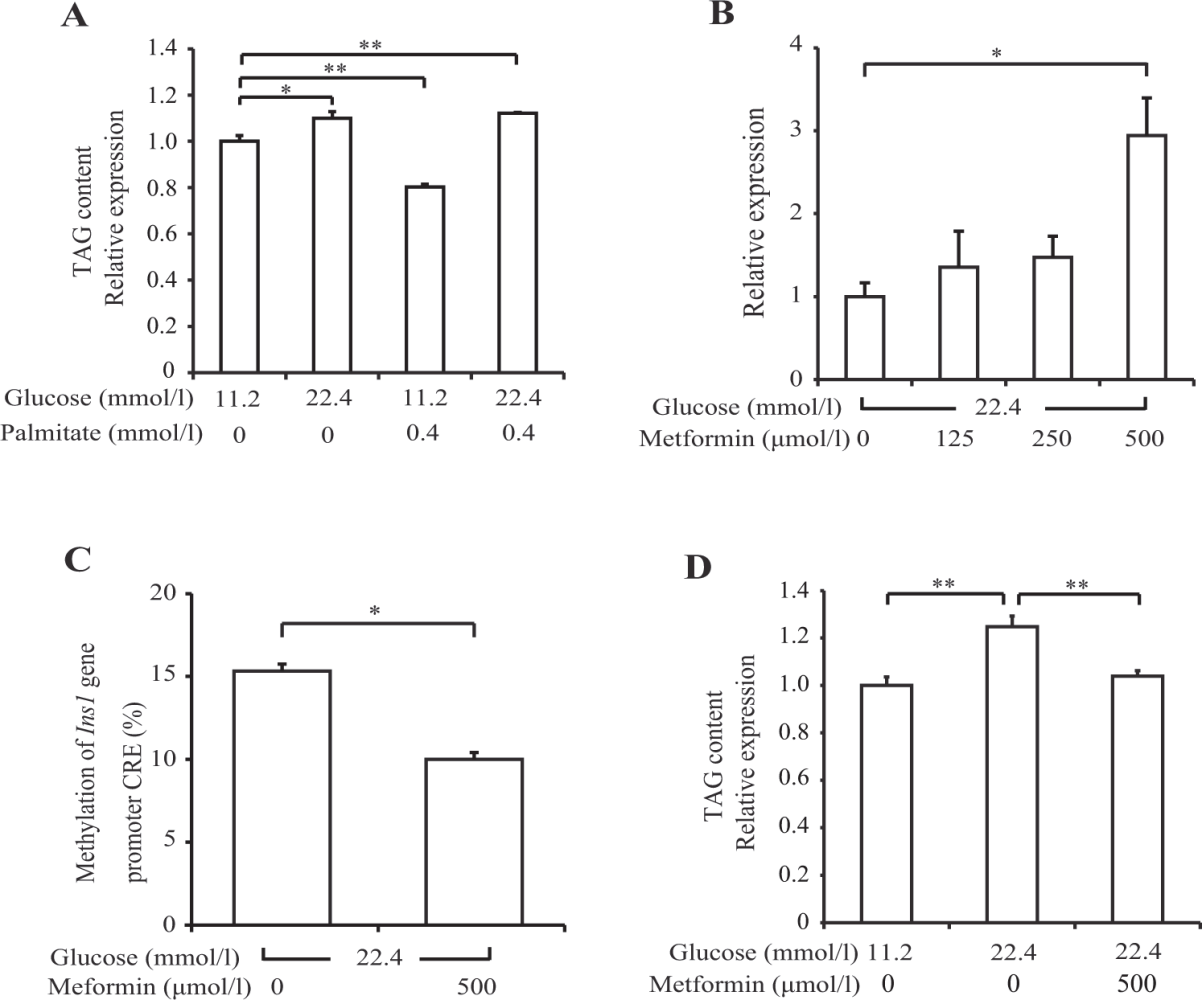
Metformin improved insulin mRNA levels, intracellular triacylglycerol (TAG) content, and DNA methylation of *Ins1* promoter. (A) INS-1 cells were cultured in glucose and palmitate for 14 days. (B-D) INS-1 cells were cultured with metformin for 14 days. Intracellular TAG levels (A and D) were examined by ELISA, insulin mRNA levels (B) were examined by real-time PCR, and DNA methylation of the *Ins1* promoter (C) was examined by pyrosequencing. All results are means ± SEM (*n* ≥ 4). Asterisks indicate statistically significant differences (*p < 0.05, **p < 0.01).

Because neither isolated oxidative stress nor ER stress affected DNA methylation, we focused on other mechanisms of glucotoxicity. Intracellular TAG accumulation was significantly increased under 22.4 mmol/l HG conditions, with and without 0.4 mmol/l palmitate, compared with that under NG conditions for 14 days in INS-1 cells (*p* < 0.05; Fig. 5A). Interestingly, intracellular TAG accumulation and DNA methylation of the *Ins1* promoter increased only under HG conditions. In addition, it is known that AMP-activated protein kinase (AMPK) activation ameliorates intracellular TAG accumulation. Therefore, we assessed the effect of metformin, which activates AMPK, on insulin mRNA levels and DNA methylation of the *Ins1* promoter. Compared with the 22.4 mmol/l HG conditions alone, metformin significantly increased insulin mRNA levels by 2.5-fold, ameliorated intracellular TAG accumulation, and decreased the DNA methylation of the *Ins1* promoter (HG: 15.3% ± 0.4%; HG plus metformin: 10.0% ± 0.7%) (Fig. 5B, 5C, 5D). These data indicate that metformin directly affects beta cells and that it inhibits the glucotoxicity-induced insulin mRNA reduction and DNA methylation of the *Ins1* promoter.

### DNA methylation of the *Ins1* promoter of the pancreatic islets of ZDF rats

**Fig 6:**
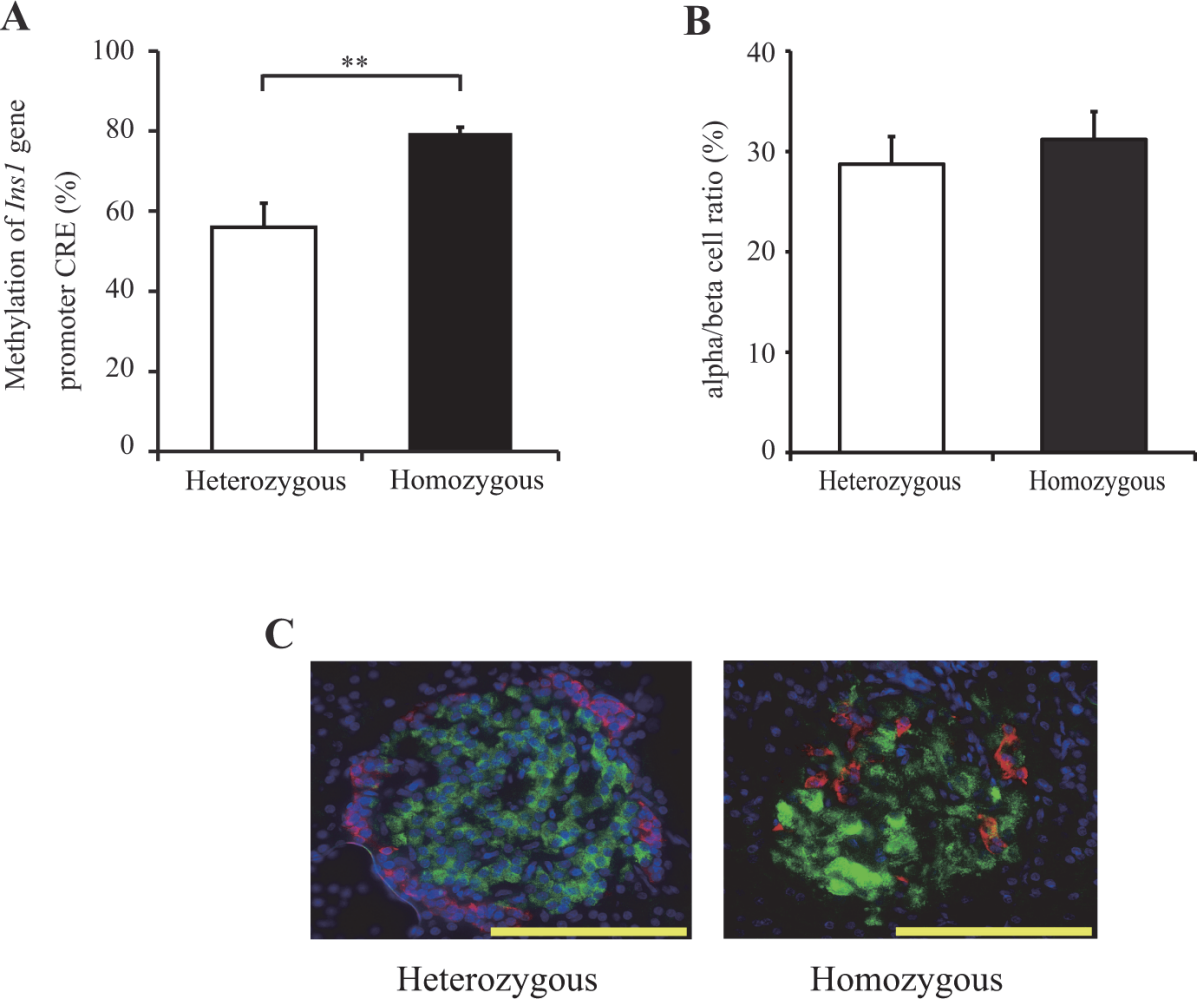
DNA methylation of *Ins1* promoter in pancreatic islets from male Zucker diabetic fatty (ZDF) rats. (A) DNA methylation of the *Ins1* promoter was examined by pyrosequencing analysis in the pancreatic islets isolated from 12-week-old ZDF rats. (B) The alpha/beta cell ratio was calculated in islets isolated from heterozygous and homozygous ZDF rats. (C) Isolated pancreases were immunostained for insulin (green), glucagon (red), and DAPI (blue) in heterozygous and homozygous ZDF rats. Scale bars indicate 100 μm. Results are mean ± SEM. A: *n* = 4 rats. B: *n* = 90 islets from 3 rats per group. Asterisks indicate statistically significant differences (*p < 0.05, **p < 0.01).

To confirm DNA methylation of the *Ins1* promoter under obese and diabetic in vivo conditions, we examined pancreatic islets from 12-week-old ZDF rats. Casual blood glucose levels increased from 8 weeks of age in ZDF homozygous (fa/fa) rats, their excessive insulin secretion gradually decreased over time, and insulin content was much lower in pancreatic islets from 12-week-old ZDF homozygous (fa/fa) rats (S3 Fig.). Furthermore, at 12 weeks, compared with nondiabetic, heterozygous (fa/+) rats, DNA methylation of the *Ins1* promoter increased (fa/+, 56% ± 6.0%; fa/fa, 79.5% ± 1.5%; *p* < 0.01) (Fig. 6A). Immunohistochemistry revealed that the alpha/beta cell ratio in islets was not significantly different between fa/fa and fa/+ individuals (fa/+, 28.7%; fa/fa, 31.2%; Fig. 6B and 6C). This result supports our in vitro experiments in INS-1 cells.

## Discussion

Our results showed that long-term exposure of pancreatic beta cells to the HG state but not to the high-fatty-acid state increased DNA methylation of the *Ins1* promoter in both time-dependent and concentration-dependent manners. To our knowledge, this is the first report to elucidate the effect of over-nutrition on DNA methylation of the *Ins1* promoter in beta cells.

Insulin gene expression and insulin secretion decrease as type 2 diabetes progresses [21,22]. In this study, insulin mRNA levels were significantly suppressed by HG incubation, and the actual transcriptional activity of the insulin gene may have been suppressed to a lesser degree than insulin mRNA levels because the HG conditions prolong the half-life of insulin mRNA [23]. Philippe et al. have shown that a 2-bp mutation (CG > TT) in CRE of rat *Ins1* resulted in a significant suppression of the gene promoter activity, indicating that the CRE site in the insulin promoter is important for insulin gene transcription [24]. Moreover, Kuroda et al. reported that DNA methylation of the CpG site in CRE of the mouse *Ins2* promoter significantly suppressed promoter activity by approximately 50% [25]. Our data revealed that HG conditions resulted in DNA methylation of the CpG site within the *Ins1* promoter and that methylation suppressed the transcriptional activity of *Ins1*. This suggests that glucotoxicity causes DNA methylation in pancreatic beta cells and that this epigenetic mechanism may be a cause of the irreversible decline in insulin mRNA levels induced by glucotoxicity.

Although this study showed that glucotoxicity increased DNA methylation by approximately 10% in INS-1 cells and that DNA methylation certainly suppressed the transcriptional activity in reporter assays, other glucotoxicity mechanisms should also be involved in the decline in insulin gene expression. In particular, the decrease in insulin gene expression at day 3 was probably caused by glucotoxicity but not DNA methylation. For example, glucotoxicity is thought to cause oxidative stress and ER stress. Oxidative stress suppresses insulin gene transcription by PDX-1 translocation from the nucleus to the cytosol by activating the cJun N-terminal kinase (JNK) pathway [26]. In addition, glucotoxicity reportedly damages the DNA binding affinity of PDX-1 [27], implying that DNA methylation is involved. The association between DNA methylation and oxidative stress has frequently been reported in cancer research [28,29]; for example, oxidative stress leads to DNA methylation of the glutathione S-transferase pi 1 gene promoter by the recruitment of transcriptional repressor complexes, including DNMTs, in prostate cancer [28]. However, a single oxidative stress load did not increase DNA methylation in our study, suggesting that oxidative stress is either irrelevant to DNA methylation or that additional factors are required in beta cells. Meanwhile, ER stress is reported to induce histone modification, such as histone H3 lysine 4 monomethylation in the monocyte chemoattractant protein-1 gene promoter, by activation of histone methyltransferase SET7/9 [30]. However, direct induction of DNA methylation by ER stress has not been reported, and our data demonstrated that a single ER stress load did not increase DNA methylation in beta cells.

In this study, DAC increased the insulin gene expression; however, the amelioration was only partial, especially compared to the level at NG. Although it was difficult to use a higher concentration of DAC and for a longer period, the partial amelioration may be caused by the decrease in DNA methylation. In addition, we assume that the accumulation of the partial amelioration results in the mitigation of progressive pathophysiology of type 2 diabetes.

In this study, HG conditions for 14 days increased *Dnmt1* mRNA levels and DNMT activity in beta cells. Our data are consistent with a previous report that showed that exposure to 16.7 mmol/l glucose for 3 days increased the *Dnmt1* mRNA level and DNA methylation in the *Pdx1* promoter in beta cells [31]. Metabolites from the methionine cycle, particularly the ratio of S-adenosylmethionine (SAM) to S-adenosyl homocysteine (SAH), and the expression of DNMT are important in the formation of methylated DNA because SAM is a donor of methyl groups for DNMTs [32]. It has been reported that in human hepatocellular carcinoma cell lines, HG conditions significantly increased DNMT activity, the ratio of SAM to SAH, and global DNA methylation [33]. Besides, the level of SAM and global DNA methylation have been significantly increased in the livers of ZDF rats [34,35]. Our data support these data, demonstrating elevated DNA methylation levels in the beta cells of ZDF rats, which suggests that altered methionine metabolism in HG conditions is involved in epigenetic changes through DNMT activity regulation in beta cells. This study also demonstrated that HG conditions suppressed TET activity without changing *Tet* mRNA levels. The activity of TET is dependent on the level of αKG, which is a cofactor in the demethylation of TET [36]. The activity of isocitrate dehydrogenase, which converts isocitrate into αKG, is reportedly suppressed by interleukin-1β [37], which is endogenously produced in beta cells during glucotoxicity [37,38]. Therefore, glucotoxicity-induced interleukin-1β overproduction may be involved in the decline in αKG accumulation and TET activity in beta cells. However, future investigation is required to elucidate the association between epigenetic modifications and the metabolic status of the methionine cycle and glucose in beta cells under long-term HG conditions.

Interestingly, the pattern of ectopic TAG accumulation in an over-nutrition state was very similar to that of DNA methylation in the beta cells. Continuous HG conditions resulted in ectopic TAG accumulation by altering the activity of lipogenic enzymes [39]. Metformin, which activates AMPK, ameliorates ectopic TAG accumulation by inhibiting acetyl-CoA carboxylase [40]. In this study, metformin decreased both ectopic TAG accumulation and DNA methylation of the *Ins1* promoter and increased insulin mRNA. Although the association between DNA methylation and ectopic TAG accumulation remains unclear, our data suggest that insulin gene upregulation by metformin is implicated in any interaction.

The present study has some limitations. First, because we could not mimic the long-term high glucose condition in Wistar rats using continuous glucose infusion or isolated islets, we used rat insulinoma INS-1 cells for the analysis of DNA methylation; these cells are known to have aberrant growth regulation and are different from primary cells. DNA methylation of the *Ins1* promoter was significantly increased in the islets of ZDF rats, and the rate of DNA methylation was much higher in the rat islets than in the INS-1 cells. This difference may have been due to the different period of high glucose load and the presence of nonbeta cells in pancreatic islets, in which the CpG cites within the insulin promoter could have been completely methylated [25]. However, considering that the alpha/beta cell ratio was comparable between heterozygous and homozygous ZDF rats in this study, the higher methylation rate in islets from homozygotes certainly indicated elevated DNA methylation of the *Ins1* promoter in beta cells. Further investigation using diabetic animal models is required to clarify the mechanism of epigenetic modification in type 2 diabetes.

Second, we cannot deny the possibility that HG worked in favor of the survival of the group containing the hypermethylated *Ins1* promoter. Further investigations using more homogeneous cell lines are required.

Finally, we performed palmitate treatment in complete INS-1 medium containing 10% FBS in the present study. Nevertheless, unsatured fatty acids present in the serum may mask the palmitate effect under such high FBS conditions. We confirmed the increase in the expression of ER stress markers such as binding immunoglobulin protein (*Bip*) and spliced X box-binding protein-1 (*Xbp-1*) by real-time PCR (S4 Fig.) and found that GSIS was significantly impaired (Fig. 1I) without changing cell viability (S4 Fig.), indicating that the palmitate treatment in this study showed a certain level of lipotoxicity. However, considering the possibility of attenuated lipotoxicity under 10% FBS culture, it is difficult to conclude that lipotoxicity was not at all involved in DNA methylation.

In conclusion, the present study provides a novel insight into the impact of glucotoxicity on beta cell epigenetics. Glucotoxicity but not lipotoxicity induced DNA methylation of the *Ins1* promoter, indicating that the accumulation of DNA methylation under prolonged HG conditions is at least implicated in the irreversible pathophysiology of diabetes. Furthermore, early treatment to normalize the glycemic profile is critical to prevent the progressive deterioration of beta cells and later diabetic complications. In the future, epigenetic modification of beta cells may represent a useful therapeutic target to prevent the progression of diabetes. Interestingly, we also identified a potential novel effect of metformin on insulin gene expression through epigenetic modification. Further investigation is required to elucidate the mechanisms underlying these epigenetic modifications in beta cells.

## Supporting Information

S1 Fig Rat *Ins1* promoter and *Irs2* promoter sequences. (A) Rat *Ins1* promoter sequence. (B) Rat *Irs2* promoter sequence. Large letters indicate CpG site. Underline indicates CRE site.
(EPS)

S2 Fig *Irs2* mRNA levels and DNA methylation of CRE site in *Irs2* promoter under high-glucose conditions. INS-1 cells were cultured under the indicated conditions for 14 days. *Irs2* mRNA levels (A) were examined by real-time PCR analysis. DNA methylation of the CRE in the *Irs2* promoter (B) was examined by pyrosequencing analysis. All results are means ± SEM (*n* ≥ 4).
(EPS)

S3 Fig Metabolic profile in Zucker diabetic fatty (ZDF) rats. (A) Random blood glucose levels in heterozygous (fa/+) (white circle) and homozygous (fa/fa) (black circle) ZDF rats aged 6–14 weeks old. Blood glucose was measured from 10:00 to 14:00. (B) Plasma insulin levels were examined in fa/+ (white bar) and fa/fa (black bar) ZDF rats aged 6–14 weeks by ELISA. (C) The insulin content of isolated pancreas from 12-week-old ZDF rats was measured. All results are means ± SEM (*n* ≥ 4). Asterisks indicate statistically significant difference (*p < 0.05, **p < 0.01).
(EPS)

S4 Fig Palmitate inducible toxicity under 10% FBS culture. INS-1 cells were cultured in 11.2 mmol/l glucose conditions with palmitate for 14 days. (A) *Bip* and (B) *Xbp-1* mRNA levels were examined by real-time PCR analysis. (C) Cell viability was examined by MTT assay. All results are means ± SEM (*n* ≥ 4). Asterisks indicate statistically significant difference (*p < 0.05, **p < 0.01).
(EPS)

S1 Table Sequences of real-time PCR primer sets. (PDF)

S2 Table Summary of bisulfite PCR and pyrosequencing primer sets. (PDF)

S3 Table Sequences of bisulfite sequencing PCR primer set. (PDF)
