# Supplementary material for: Long-Term Pancreatic Beta Cell Exposure to High Levels of Glucose but Not Palmitate Induces DNA Methylation within the Insulin Gene Promoter and Represses Transcriptional Activity
Source: PLoS One. 2015 Feb 6;10(2):e0115350. doi: 10.1371/journal.pone.0115350 (PMC4319953; doi:10.1371/journal.pone.0115350)
Supplement: S1 Table — (PDF) [file pone.0115350.s005.pdf]

Table S1 Sequences of real-time PCR primer sets.

Gene (Gene ID)	Forward Primer	Reverse Primer
Rat *Ins1* (24505)	5' -GTGGGGAACGTGGTTTCTT- 3'	5' -GCAGTAGTTCTCCAGTTGGTAGAGG - 3'
Rat *Irs2* (29376)	5' -CCCCAGTGTCCCCATCCT- 3'	5' -TTTCCTGAGAGAGACGTTTTCCA- 3'
Rat *Dnmt1* (84350)	5' -CGGCTCAAAGACTTGGAAAG- 3'	5' –TAGCCAGGTAGCCTTCCTCA- 3'
Rat *Dnmt3a* (444984)	5' -CCGGGTGCTATCTCTCTTTG - 3'	5' -TGACGATGGAGAGGTCATTG- 3'
Rat *Dnmt3b* (444985)	5' -TAGGGTCCTGTCCCTGTTTG- 3'	5' -GTGATTTTCCGGACGTCATT- 3'
Rat *Tet1* (309902)	5' -GAAACCCTGAATTGGCAAAA- 3'	5' -GGGTGAGCTTTCTGATCGAC- 3'
Rat *Tet2* (310859)	5' -CCCTCACTAGAGAAGACAATCGAG- 3'	5' -GATCCACTAACCTCCTGACTCTTC- 3'
Rat *Tet3* (680576)	5' -GAGAAGCTAAGCACACCAGAGAAG- 3'	5' -CTGCTCATACTGTAGGGGTCAGAG- 3'
Rat *Bip* (25617)	5' -CCTGTTGCTGGACTCTGTGA- 3'	5' -GAATACACCGACGCAGGAAT- 3'
Rat *spliced Xbp-1* (289754)	5' -CTGAGTCCGAATCAGGTGCAG- 3'	5' -ATCCATGGGAAGATGTTCTGG- 3'
Rat *Pi3k p85* (25513)	5' -GAGAGGAAGACATCGACCTACACT- 3'	5' -CCTCTCCCCAGTAGTTTCATTG- 3'
