# Supplementary material for: Long-Term Pancreatic Beta Cell Exposure to High Levels of Glucose but Not Palmitate Induces DNA Methylation within the Insulin Gene Promoter and Represses Transcriptional Activity
Source: PLoS One. 2015 Feb 6;10(2):e0115350. doi: 10.1371/journal.pone.0115350 (PMC4319953; doi:10.1371/journal.pone.0115350)
Supplement: S2 Table — (PDF) [file pone.0115350.s006.pdf]

Table S2 Summary of bisulfite PCR and pyrosequencing primer sets.

Gene	Primer sequences	Pyrosequencing primers	Condition
*Ins1*pro-CRE	Forward<br>5' -TGGGATAATGATTGTGTTGTGAA- 3'<br>Reverse - Universal<br>5' -U- AAAAACTAAAACTATAATTTCCAAACACTT- 3'	5' -TTAGGTTTAAGTAGAGTTGTTG - 3'	55℃ (55)
*Irs2*pro-*CRE*	Forward - Universal<br>5' -U- GTYGAAGYGGGGGGTGGTTA- 3'<br>Reverse<br>5' -RAACCCAAAAAATAAACTAAAA- 3'	5' -CCAAAAAATAAAAAAC - 3'	52.5℃ (55)

U indicates the universal primer sequence: biotin-GGGACACCGCTGATCGTTTA. Number in parentheses indicates cycles at the indicated temperature.
