# Supplementary material for: Long-Term Pancreatic Beta Cell Exposure to High Levels of Glucose but Not Palmitate Induces DNA Methylation within the Insulin Gene Promoter and Represses Transcriptional Activity
Source: PLoS One. 2015 Feb 6;10(2):e0115350. doi: 10.1371/journal.pone.0115350 (PMC4319953; doi:10.1371/journal.pone.0115350)
Supplement: S3 Table — (PDF) [file pone.0115350.s007.pdf]

Table S3 Sequences of bisulfite sequencing PCR primer set.

Gene	Forward	Reverse	Condition
Ins1 promoter	5' -GAGTTGGGGTTTTAGTTGAGTT - 3'	5' -AAAAAAAAAACCCACATCCTT - 3'	55℃ (40)

Number in parentheses indicates cycles at the indicated temperature.
